# Exploring Clinical Governance Interventions and Organisational Learning in Public Hospitals in South Africa’s Eastern Cape and Mpumalanga Provinces: A Mixed-Methods Study Protocol

**DOI:** 10.3390/healthcare13192430

**Published:** 2025-09-25

**Authors:** Kedibone Maake, Wezile Chitha, Sibusiso C. Nomatshila, Sikhumbuzo A. Mabunda

**Affiliations:** 1 Department of Public Health, Walter Sisulu University, Mthatha 5119, South Africa; wchitha@wsu.ac.za (W.C.); snomatshila@wsu.ac.za (S.C.N.); smabunda@wsu.ac.za (S.A.M.); 2 Institute for Clinical Governance and Healthcare Administration, Walter Sisulu University, Mthatha 5119, South Africa; 3 Society and Health Research Institute, Walter Sisulu University, Mthatha 5119, South Africa; 4 Global Centre for Human Resources for Health Intelligence, Walter Sisulu University, Mthatha 5119, South Africa; 5 School of Population Health, University of New South Wales, Sydney 2033, Australia; 6 George Institute for Global Health, University of New South Wales, Sydney 2033, Australia

**Keywords:** clinical governance, organisational learning, public hospitals, South Africa

## Abstract

Safeguarding patient and personnel safety and improving care quality has emerged as a critical priority for healthcare systems globally. In response to persistent challenges in healthcare delivery, many countries have adopted clinical governance frameworks and organisational learning processes to strengthen accountability and promote continuous improvement. Robust clinical governance frameworks provide the processes and accountability measures necessary to foster a culture of knowledge-sharing and evidence-based decision-making, all of which are key characteristics of a learning organisation. This study seeks to investigate the role of clinical governance in improving hospital performance through three interconnected sub-studies. The first sub-study will explore how non-clinical managers in selected public sector hospitals leverage clinical governance to improve hospital performance. The second sub-study will evaluate the impact of clinical governance interventions on clinical outcomes and identify opportunities for organisational learning within these hospitals. The third sub-study will serve as an embedded experimental component, monitoring changes in complaint resolution indicators before and after interventions to assess improvements in clinical governance through both intra- and inter-hospital comparisons. Qualitative data will be analysed using NVivo version 15, with inductive thematic analysis employed to uncover emergent patterns and interpretive themes.

## 1. Introduction

Recently, healthcare quality and patient safety discussions have prominently featured two critical concepts: clinical governance and organisational learning [1,2,3]. Clinical governance refers to the system through which healthcare providers are accountable for continuously improving the quality of their services and maintaining high standards of care [4]. Clinical governance involves a systematic approach to maintaining and improving the quality of patient care within a healthcare setting, and its key elements include clinical effectiveness, clinical audit, risk management, continuing professional development, patient and public involvement, staff management, and information management [5]. Organisational learning describes how organisations acquire, share, and utilise knowledge to enhance performance and adaptability [1]. These frameworks are crucial in shaping operational efficiency and patient outcomes within health facilities, especially in public hospitals’ dynamic and challenging environments [1,5]. Furthermore, organisational learning within healthcare institutions is essential for fostering a continuous learning and education culture, one of the key pillars of clinical governance [1,5].

Globally, there is growing recognition of the need to strengthen clinical governance and promote organisational learning to address persistent challenges, such as poor service quality, patient safety issues, and inefficient resource utilisation [6,7,8]. In 1998, the United Kingdom (UK) introduced a clinical governance framework in response to similar challenges; they implemented the framework to address primarily a wide variation in the quality of care provided by the National Health Service (NHS) [9]. The Italian government benchmarked the clinical governance concept for their NHS from the UK and described it as a system that fulfils the needs of patients and health workers through the integration of clinical and managerial activities, guaranteeing the continuous improvement of quality concerning the principles of equity, appropriateness, and universality [2]. Canada implemented clinical governance to reduce costs and enhance the quality of care [9]. This decision was made after the Organisation for Economic Co-operation and Development (OECD) data showed that Canada was spending more per capita than its peers and experiencing poorer outcomes regarding re-admissions, longer waiting times, and increased complications. The development of clinical governance in New Zealand has been a crucial aspect of the country’s health policy since 2009 [4]. Assessments of this policy between 2010 and 2012 revealed reasonable progress in its development during that period [4].

The implementation of clinical governance in many low- and middle-income countries (LMICs), such as South Africa, is exacerbated by unique contextual challenges, including resource constraints, high disease burdens, workforce shortages, and organisational culture barriers [10]. A scoping review of health system governance interventions in LMICs revealed that evidence linking clinical governance to quality care remains fragmented, with stronger evidence supporting health financing, private sector partnerships, and community engagement strategies compared to leadership, system design, information systems, and accountability mechanisms [11]. Research from Iran revealed that hospitals’ preparedness for clinical governance implementation was typically characterised as average or weak, with the lowest scores reflecting deficiencies in a planned and integrated quality improvement program [12]. Additionally, a study conducted in the Eastern Cape and Mpumalanga provinces showed a limited awareness and inconsistent application of clinical governance protocols among healthcare staff [13]. These findings imply that the challenges associated with implementation may exhibit similar trends across various low- and middle-income countries LMICs.

Rural provinces in South Africa experience difficulties, such as a lack of infrastructure and human resources, which affect the quality of services provided, especially within public sector hospitals [14,15]. South Africa’s health system is fragmented, with 8.3% of the Gross Domestic Product (GDP) contributed by health split equally between the private sector (4.1%) and the public sector (4.2%) [14,15,16]. The private sector covers about 20% of the population, most of whom have private medical insurance, while the remaining 80% of the population, who mostly rely on the public health sector and cannot afford private medical insurance, receive a smaller share of the health expenditure [14,15,16]. This unequal distribution of resources has resulted in a disparity in infrastructure and human resources, with a bias towards the private sector [14,15,16]. Additionally, South Africa faces a quadruple burden of diseases, including violence and injuries, non-communicable diseases, communicable diseases like HIV/AIDS and tuberculosis, and high maternal and child mortality rates [14,15,16]. These challenges have a negative impact on access to overall hospital performance [14,15,16].

The South African government has taken several steps to improve healthcare services for its citizens [16]. One such initiative is the establishment of the Office of Health Standards Compliance (OHSC), which has developed the National Core Standards (NCS) as a tool for clinical governance and a national benchmark for measuring service quality [17]. Additionally, the government is implementing the National Health Insurance (NHI) to promote equity and efficiency in healthcare, ensuring that all South Africans, regardless of their socio-economic status, have access to affordable, quality healthcare services [18]. However, despite these efforts, there have been reports from communities and the media that the public health system needs to meet basic standards of care and improve the patient experience of care [14]. For instance, places like the Eastern Cape and Mpumalanga provinces in South Africa consistently demonstrate poor health outcomes despite significant interventions and investments made by the National Department of Health (NDoH) to strengthen the capacity of the health system to deliver quality healthcare services [19,20,21,22]. A recent study conducted across four hospitals in South Africa revealed that clinical staff had a limited understanding of existing clinical governance protocols and tools [13]. The understanding and implementation of clinical governance and organisational learning in public health facilities is crucial for ensuring quality healthcare provision and ensuring that lessons are drawn from previous experiences [1,5,23]. Previous research has demonstrated that robust clinical governance structures and organisational learning mechanisms are crucial for ensuring patient safety, high-quality care, and accountability in healthcare facilities [1,4]. However, there is a scarcity of comprehensive studies that focus on the status of effective clinical governance interventions and organisational learning in public sector hospitals in South Africa [10,24]. By filling this research gap, this study aims to explore effective and sustainable clinical governance interventions and organisational learning processes in two South African rural provinces. A full understanding and effective implementation of these interventions can inform policy decisions and interventions to improve healthcare quality and patient safety in South Africa and other similar settings.

## 2. Objectives

This study will answer the research objectives through three sub-studies, summarised in Table 1.

## 3. Study Setting

The study will be conducted in two South African rural provinces, Eastern Cape and Mpumalanga, which are characterised by high levels of poverty, unemployment, and socioeconomic inequality, which directly impact healthcare outcomes [21,22]. Most people rely on public health facilities for healthcare [22]. Both these provinces consistently demonstrate poor health outcomes despite significant interventions, strategies, and investments made by the Department of Health (DoH) to strengthen the capacity of the health system to deliver quality healthcare services [16,20,21]. The DoH in the Eastern Cape has faced numerous challenges, such as overcrowded hospital wards, outdated infrastructure, and lack of essential resources, which have not only been the subject of negative media attention but have also directly affected the public [22]. A report by the DoH in 2017 revealed that 13 out of the 25 hospitals in Mpumalanga were facing critical shortages of essential medical equipment, linen, beds, and utilities such as water and electricity, which posed significant challenges in providing proper care to patients [25,26]. The study setting is four (4) hospitals: two hospitals in the Eastern Cape, Nelson Mandela Academic Hospital and St Elizabeth Hospital, and two hospitals in Mpumalanga, Rob Fereira Hospital and Themba Hospital. The selected hospitals act as referral hospitals within their respective provinces, each offering different levels of specialised healthcare services. The selection of hospitals for the study utilised a simple random sampling technique to ensure an unbiased and representative sample.

## 4. Study Design

### 4.1. Sub-Studies 1 & 2

The two sub-studies will utilise a qualitative approach and will be reported using the Consolidated Criteria for Reporting Qualitative Research (COREQ) [27]. Individual, semi-structured, in-depth interviews will be conducted with both clinical and non-clinical managers of the four study hospitals before and after a 12-month intervention period [10]. The intervention involves the implementation of seven clinical governance protocols, each aligned with national and international standards for quality and safety in healthcare. These protocols are supported by a structured capacity-building program that includes targeted training sessions and on-site coaching for hospital managers and healthcare professionals. The training will be delivered by experienced clinical governance facilitators and will focus on operationalising the protocols within existing hospital workflows. On-site coaching will provide ongoing support, facilitate problem-solving, and reinforce adherence to the protocols throughout the intervention period [10]. The intervention is part of a broader study where seven protocols are implemented at the study sites to determine effectiveness in improving clinical governance [10,13,28]. The seven protocols are:

Clinical Governance

Complaint management

Clinical guidelines

Clinical audits

Education and training

Infection and prevention control

Patient safety incident management

Two hospitals will serve as intervention sites (St Elizabeth Hospital and Themba Hospital), and two will serve as non-intervention sites (Nelson Mandela Academic Hospital and Rob Ferreira Hospital).

These sub-studies will include the hospital board chairperson, Chief Executive Officer (CEO), corporate services manager, clinical managers, quality assurance managers, nursing services managers, and the provincial representative responsible for overseeing clinical governance activities. The aim is to evaluate their understanding of clinical governance and the systems in place to monitor the implementation of effective and current clinical governance practices, before the intervention (sub-study 1) and after the intervention (sub-study 2).

### 4.2. Sub-Study 3

Sub-study 3 will utilise an embedded experimental aspect of the study to assess changes in clinical governance through both intra-hospital and inter-hospital comparisons, with a specific emphasis on the complaints management protocol, and will be reported on using the Consolidated Standards of Reporting Trials (CONSORT) [29]. Complaints management is a critical aspect of clinical governance, and assessing changes in complaint resolution metrics will offer tangible evidence of improvements in patient care and organisational responsiveness [30]. Handling of complaints can reflect an institution’s leadership and learning culture. Complaint resolution data are routinely recorded in hospital management systems, making this information relatively accessible [30]. Intra-hospital comparisons will evaluate the performance of intervention hospitals by utilising complaint resolution indicators based on outcomes prior to and following the intervention. Inter-hospital comparisons will compare the performance of the intervention hospitals with that of the non-intervention hospitals on complaint resolution. By examining both intra- and inter-hospital data, the study provides a comprehensive view of the intervention’s effectiveness.

The sub-study will monitor changes in key complaint resolution indicators before and after a 12-month intervention conducted within the intervention hospitals. The sub-study will adopt a comparative approach, analysing four specific indicators from the complaints management protocol:

1. Total number of complaints received within a month—The raw count of formal, written complaints received by the hospital’s complaints management unit per month.
2. Complaint resolution rate—The proportion of resolved complaints to total complaints received within a given month, expressed as a percentage.
3. Complaint resolution within 25 working days—The proportion of complaints resolved within ≤25 working days of receipt to total complaints received in a month, expressed as a percentage.
4. Severity of complaints—Complaints will be categorised using a validated severity classification framework. The monthly proportion of complaints in each category will be calculated.

These indicators will be assessed over a three-month period before the intervention (from 1 January to 31 March 2024) and after a 12-month intervention where seven clinical governance protocols are currently being implemented in another study [10]. The four complaint management indicators are quantifiable, relevant to policy, and directly linked to patient experience and institutional accountability.

#### 4.2.1. Sample Size Estimation and Data Structure

Continuous monthly time series will be utilised for each of the four indicators across all participating hospitals. The data will cover a pre-intervention period of three months and a post-intervention period of three months, resulting in a total of six data points for each hospital per indicator. With four hospitals involved, this gives 24 observational units for pre-post analysis. While the limited number of data points restricts the use of complex time-series models, it is adequate for preliminary trend analysis. Additionally, the data will be treated as a panel dataset (longitudinal data across multiple hospitals), allowing us to utilise statistical models that account for variations both within and between hospitals.

#### 4.2.2. Statistical Analytical Approach

Data will be analysed using Stata 19.5. The Shapiro–Wilk test will be used to test the normality of the distribution of numerical variables. Numerical data will be summarised using the mean, standard deviation (SD), and range if normally distributed. The median and interquartile range will be used to summarise numerical data that are not normally distributed. The paired t-test or the Wilcoxon signed rank test will be used to compare pre- and post-intervention numerical data, depending on the normality of the distribution. Categorical data will be summarised using frequencies, percentages, and graphs. The McNemar’s test will be used to compare categorical data before and after the intervention, stratified by hospitals. Inter and intra-hospital variations will be compared.

For proportions, such as resolution rates, the chi-square tests for trend analysis of grouped monthly data will be utilised.

Time-Series: Where monthly data over the entire period are available, segmented regression will evaluate changes in level and slope of the outcomes following the intervention, providing robust estimation while controlling serial autocorrelation. The level of significance will be a *p*-value ≤ 0.05.

## 5. Population and Sampling

### Sub-Studies 1 & 2

The study will involve the purposive recruitment of managers within the healthcare system, specifically targeting individuals in prominent leadership roles. This includes all chairpersons of hospital boards, Chief Executive Officers (CEOs), managers of corporate services, clinical managers, quality assurance managers, nursing services managers, and provincial representatives responsible for overseeing clinical governance activities within the four study hospitals. The number of participants will depend on the number of managers in each hospital. Each participant will engage in comprehensive individual interviews that are semi-structured and in-depth, allowing for a thorough exploration of their perspectives and experiences.

To ensure active participation and sustained engagement from hospital stakeholders, the study will adopt a participatory approach based on the principles of co-design and shared ownership. Key stakeholders, including the Chief Executive Officer, Clinical Manager, Nursing Manager, Corporate Manager, Hospital Board Manager, and Quality Assurance Manager, will be involved from the beginning of the study through structured workshops and feedback sessions. To minimise disruption and reduce the burden on staff, the intervention has been designed to integrate seamlessly into existing clinical workflows. After completion of the study, the researchers will work with the Executive Committee of the hospital to co-develop a straightforward sustainability plan. This plan will include a formal handover of all project materials, tools, and data to the Quality Assurance Unit, along with a discussion on how to incorporate successful intervention components into standard operating procedures and future operational plans.

## 6. Data Collection

### Sub-Studies 1 & 2

An in-depth interview guide will be employed to collect data from the non-clinical management of hospitals. These interviews are designed to elicit significant perceptual insights from participants, allowing them to articulate their views in their own words. The interview guide will encourage discussions on topics that may be challenging to explore through a structured questionnaire. Specifically, it will address areas such as challenges facing hospitals; perceptions of hospital performance; performance indicators; understanding of clinical governance; the existence of clinical quality improvement or clinical governance plans; the implementation status of these plans; systems for executing key clinical governance and quality improvement activities; current structures that support the enforcement of these initiatives; as well as barriers and facilitators to their implementation. With the participant’s consent, an audio recorder will be used to capture the interviews. Interview data will be transcribed verbatim.

## 7. Data Management and Analysis

### Sub-Studies 1 & 2

Qualitative data will be analysed to uncover meaningful and symbolic content using NVivo version 15. This process will involve inductive thematic analysis [31], allowing the research findings to emerge from the predominant themes identified in the raw data. The emerging findings will be summarised using descriptive words, phrases, themes, or patterns to facilitate a better understanding and interpretation of these concepts. Furthermore, the analysed data will be contextualised within the framework of existing literature, highlighting how it aligns with current knowledge or provides new insights into the established body of work.

## 8. Trustworthiness of the Study

To enhance the trustworthiness of the study, strategies such as member checking, thick description, maintaining an audit trail, reflexivity, and conducting peer debriefing will be incorporated [32]. These strategies will strengthen the rigor and reliability of the study, ensuring that the findings are credible, transferable, dependable, and confirmable.

Credibility refers to the confidence in the truth of the findings and the extent to which the study accurately represents the participants’ perspectives and experiences [32]. The utilisation of semi-structured, in-depth interviews allows for a detailed exploration of participants’ perspectives, enabling the researcher to capture rich, nuanced data. The focus on key leadership roles ensures that participants have direct experience and knowledge of clinical governance and its impact on hospital performance. However, employing a convenience sampling method may introduce selection bias, potentially affecting the study’s credibility. To mitigate this, the researcher will use member checking, where participants will review and validate the findings, ensuring an accurate representation of their views.

Transferability refers to the extent to which the findings can be applied to other contexts [32]. This study provides a detailed description of its setting, which is public sector hospitals in the Eastern Cape and Mpumalanga, and the participants, who are non-clinical managers in leadership roles, which allows readers to assess the applicability of the findings to similar settings. The focus on clinical governance and its role in enhancing hospital performance is relevant for healthcare systems globally, especially in resource-constrained settings. However, the use of convenience sampling and the focus on only four hospitals may limit the generalisability of the findings to other provinces or countries. The researcher aims to provide a thick description of the context, participants, and findings to enable readers to determine the study’s relevance to their own settings.

Dependability refers to the consistency and stability of the findings over time and across different researchers [32]. To establish dependability, the researcher will provide a comprehensive and detailed account of the study’s design, along with the data collection and analysis procedures. This transparency will enable others to replicate the study if they choose to do so. The utilisation of semi-structured interviews with a clear interview guide will ensure that data collection is systematic and consistent across participants. Additionally, the researcher will maintain a detailed audit trail, including notes on data collection, coding, and analysis, to ensure transparency and reproducibility.

Confirmability refers to the objectivity of the findings, ensuring they are shaped by the participants’ experiences rather than influenced by researcher bias [32]. Utilising in-depth interviews enables participants to articulate their perspectives freely, thereby minimizing the impact of the researcher’s assumptions. Additionally, the researcher will engage in reflexivity by maintaining a journal to record their assumptions, biases, and decision-making processes throughout the study.

Reflexivity refers to the analytical attention given to the role of the researcher in qualitative research [33]. It entails a continuous process of self-critique and self-appraisal, wherein the researcher articulates how their personal experiences may have influenced or not influenced various stages of the research process. For this study, the research team consists of health professionals who are both knowledgeable and passionate about clinical governance. Engaging in reflexivity is crucial to mitigate the potential impact of the researchers’ knowledge and unconscious biases on their perspectives throughout the research process, including the framing of questions and responses to participants’ answers. Prior to initiating data collection, the research team will participate in collective reflection during one of their training sessions to discuss their expectations and assumptions within the context of the research.

## 9. Ethics and Dissemination

Ethical clearance was obtained from the Research Ethics Committee of the Faculty of Health Sciences at Walter Sisulu University, Ref: WSU HREC 051/2025. Approval to access the research sites was obtained from both the Provincial Health Research Committees of the Eastern Cape and the Mpumalanga Department of Health. Before data collection, entry to the study sites will be further negotiated with the hospital CEOs. The study will abide by the 4 ethical principles of autonomy, beneficence, non-maleficence, and justice.

Information sheets will be provided to all participants before the in-depth interviews and structured questionnaires. Informed consent forms will also be signed by all study participants, including consent for audio recordings of the interviews. The consent forms will be thoroughly explained, detailing the study’s purpose, objectives, participant expectations, and any associated risks and benefits of participation. If a participant chooses not to be recorded, a second option will be available to take notes manually. Participants will be assured that their involvement in this study is entirely voluntary and that their confidentiality will be upheld throughout the research process.

Participants will also be assured that they can withdraw from the study at any time and choose not to answer any questions they find uncomfortable without facing any negative consequences. All identifying information will be removed, including data collected from audiotapes during one-on-one interviews. Additionally, audiotapes will be transferred to a password-protected computer, after which the recordings will be deleted from the tape recorder. Once the audiotape has been transcribed, the audio recording will be destroyed to ensure confidentiality. All electronic records will be encrypted with a password. Only the researchers will have access to the data.

Research findings will be communicated through several dissemination mechanisms, including workshops, media, seminars, and conferences, and published in peer-reviewed journals. This will ensure that the research findings reach the right stakeholders and can be utilised to inform decisions, policy, and further research.

## 10. Discussion and Expected Outcomes

This study protocol evaluates clinical governance interventions and organisational learning within public sector hospitals. It examines the role of hospital managers in utilising clinical governance frameworks to enhance hospital performance, thereby addressing a significant gap in the literature regarding health systems leadership and quality improvement in LMICs. The anticipated outcomes include a deeper understanding of how clinical governance frameworks are implemented in resource-constrained environments, the identification of effective interventions that foster organisational learning, and insights into the contextual factors that affect the success of these implementations.

Additionally, the study will assess the extent to which existing clinical governance interventions contribute to improved hospital efficiency and service quality. The protocol’s potential contribution is to provide insights into whether these managers effectively bridge the gap between administrative decision-making and clinical service delivery. The research will explore potential challenges that hospital managers face when implementing clinical governance. Additionally, it will assess the extent to which existing clinical governance interventions contribute to enhanced hospital efficiency and service quality. The findings may reveal gaps in clinical governance implementation, such as inconsistent monitoring, insufficient resource allocation, or weak accountability mechanisms. Furthermore, the study will thus explore best practices in clinical governance that have led to measurable improvements in key performance indicators, reduced patient waiting times, and increased staff morale. These findings are expected to inform policy and practice not only in South Africa but also in other regions facing similar systemic challenges.

## 11. Study Limitations

This study examines the public healthcare systems in Eastern Cape and Mpumalanga provinces. We acknowledge that these areas possess distinct sociocultural demographics, historical contexts, governance structures within provincial health departments, and resource limitations. In addition, Mpumalanga province has no medical school, while there is a medical school in Eastern Cape province. These contextual factors are crucial for understanding how clinical governance interventions are implemented and their subsequent outcomes. As a result, the findings will be inherently influenced by this specific context. The primary implication is that the results should not be indiscriminately generalised to all South African provinces or to different global settings. Instead, the true value of this research lies in its potential to offer detailed, contextually relevant insights into the mechanisms and processes that contribute to either successful or unsuccessful clinical governance and organisational learning within under-resourced public health systems. Furthermore, the study has a primarily observational nature. Future researchers, informed by the mechanisms and theories generated from this exploratory study, would benefit from incorporating more robust causal inference techniques, such as quasi-experimental designs or mixed-method longitudinal analyses. These methods could further validate and extend the insights generated by our study.

To enhance transferability and address potential limitations, the study will implement several strategies. A detailed contextual description of the results, highlighting socioeconomic, cultural, and systemic factors, will be provided. This approach will assist healthcare providers in assessing the relevance of the findings to their own settings. Additionally, data collection will focus on creating a comprehensive depiction of the context, including thorough accounts of the hospital environments, participant profiles, institutional histories, and the specific challenges and facilitators encountered. Such detail will enable healthcare providers to evaluate how closely their own contexts align with the study sites and determine the applicability of the findings. Negative or null results will not be seen as a failure of the clinical governance concept; instead, they should be viewed as important data reflecting implementation failures or contextual barriers. Inconclusive outcomes will be interpreted as indicators of the intervention’s sensitivity to context or potential methodological limitations. Furthermore, the study will employ established theoretical frameworks, such as organisational learning theory and clinical governance frameworks. This will create a common language and conceptual structure, ensuring that the conclusions can be understood and applied, even if the specifics differ. By doing so, the results of the study will assist researchers and policymakers in various settings to interpret the findings through a shared perspective, promoting cross-contextual comparison and application.

## Figures and Tables

**Table 1:** Sub-studies and their related objectives.

Sub-Studies	Objectives
Sub-study 1	To investigate how non-clinical managers in public sector hospitals in the Eastern Cape and Mpumalanga provinces of South Africa utilise clinical governance to enhance hospital performance.
Sub-studies 2 and 3	To investigate the effectiveness of clinical governance interventions and opportunities for organisational learning in selected public hospitals within South Africa’s Eastern Cape and Mpumalanga provinces, focusing on improving clinical outcomes and organisational learning.

## Data Availability

Data is contained within the article. The original contributions presented in this study are included in the article. Further inquiries can be directed to the corresponding author.

## Abbreviations

The following abbreviations are used in this manuscript:

UK	United Kingdom
NHS	National Health Service
LMIC	Low- and middle-income countries
GDP	Gross Domestic Product
OHSC	Office of Health Standards Compliance
NCS	National Core Standards
NHI	National Health Insurance
NDoH	National Department of Health
CEO	Chief Executive Officer
